# Space-occupying brain lesions, trauma-related tau astrogliopathy, and ARTAG: a report of two cases and a literature review

**DOI:** 10.1186/s40478-021-01152-3

**Published:** 2021-03-23

**Authors:** Adam D. Bachstetter, Filip G. Garrett, Gregory A. Jicha, Peter T. Nelson

**Affiliations:** 1 Spinal Cord and Brain Injury Research Center, University of Kentucky, 741 S. Limestone St., Lexington, KY 40536 USA; 2 Department of Neuroscience, University of Kentucky, Lexington, KY USA; 3 Sanders-Brown Center on Aging, University of Kentucky, Lexington, KY USA; 4 Department of Pathology and Laboratory Medicine, University of Kentucky, Lexington, KY USA; 5 Department of Neurology, University of Kentucky, Lexington, KY USA

**Keywords:** Tangles, NFTs, PSP, TBI, Tauopathy, Meninges, FTLD

## Abstract

Astrocytes with intracellular accumulations of misfolded phosphorylated tau protein have been observed in advanced-stage chronic traumatic encephalopathy (CTE) and in other neurodegenerative conditions. There is a growing awareness that astrocytic tau inclusions are also relatively common in the brains of persons over 70 years of age—affecting approximately one-third of autopsied individuals. The pathologic hallmarks of aging-related tau astrogliopathy (ARTAG) include phosphorylated tau protein within thorn-shaped astrocytes (TSA) in subpial, subependymal, perivascular, and white matter regions, whereas granular-fuzzy astrocytes are often seen in gray matter. CTE and ARTAG share molecular and histopathologic characteristics, suggesting that trauma-related mechanism(s) may predispose to the development of tau astrogliopathy. There are presently few experimental systems to study the pathobiology of astrocytic-tau aggregation, but human studies have made recent progress. For example, leucotomy (also referred to as lobotomy) is associated with a localized ARTAG-like neuropathology decades after the surgical brain injury, suggesting that chronic brain injury of any type may predispose to later life ARTAG. To examine this idea in a different context, we report clinical and pathologic features of two middle-aged men who came to autopsy with large (> 6 cm in greatest dimension) arachnoid cysts that had physically displaced and injured the subjects’ left temporal lobes through chronic mechanical stress. Despite the similarity of the size and location of the arachnoid cysts, these individuals had dissimilar neurologic outcomes and neuropathologic findings. We review the evidence for ARTAG in response to brain injury, and discuss how the location and molecular properties of astroglial tau inclusions might alter the physiology of resident astrocytes. These cases and literature review point toward possible mechanism(s) of tau aggregation in astrocytes in response to chronic brain trauma.

## Introduction

Much remains unknown about astrocytes in healthy and diseased states. Here we discuss a specific subset of astrocyte pathology, related to a hypothesis that localized trauma can induce tau astrogliopathy, which is similar in appearance to age-related tau astrogliopathy (ARTAG). It has recently become clear that chronic traumatic encephalopathy (CTE) is associated with both neuronal and glial tau pathology [1, 2]. Further, even decades after neurosurgical procedures severing frontal white matter tracts (leucotomies), the frontal cortical tissue that was traumatically affected tended to contain astrocytes with abnormal tau accumulations [3]. These observations raised intriguing questions about human astrogliopathy seen commonly at autopsy, and the inter-relationships between brain trauma and astrocytic tau proteinopathy. This review includes a presentation of two cases that experienced chronic mechanical stress induced by large, localized, space-occupying brain lesions (arachnoid cysts), examined for ARTAG and other pathologies. These case reports are followed by a broader discussion of relevant scientific literature, focusing on human studies.

## Case reports

Arachnoid cysts are non-neoplastic alterations of the leptomeninges resulting in a fluid-filled space between the brain or spinal cord and the arachnoid membrane. Arachnoid cysts usually arise from arachnoid membrane splitting in early development, although secondary cysts have been reported following head trauma [4, 5]. In adults, arachnoid cysts are often clinically silent and may persist for decades [6, 7]. We present clinical and pathologic findings from two adult males with large arachnoid cysts. Note that in keeping with terminology applied in other conditions [8, 9], we distinguish between ARTAG (concept and disease) and the relevant neuropathologic hallmarks (ARTAG–NC); likewise for CTE and CTE–NC.

**Fig. 1:**
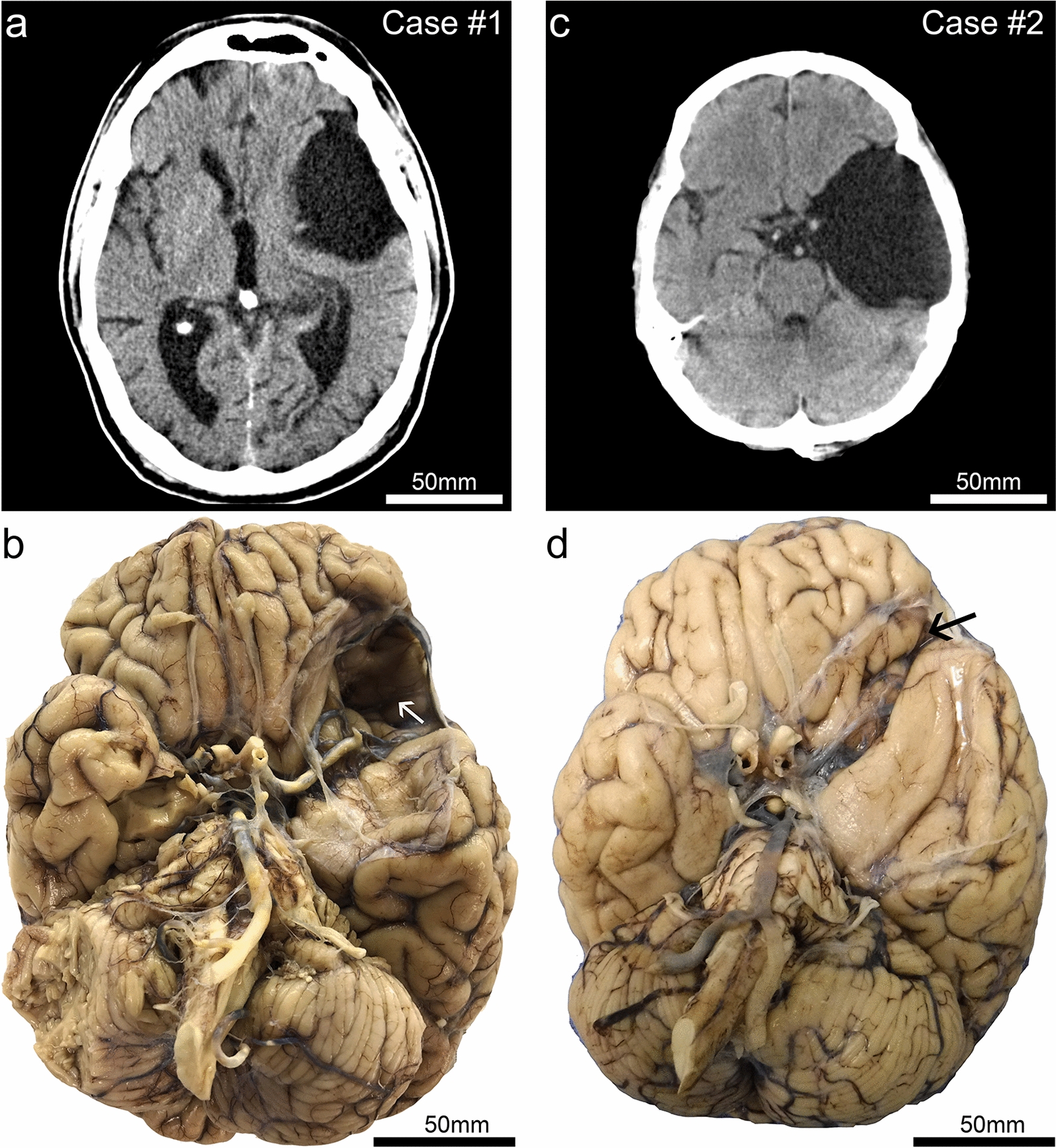
Clinical (premortem) computerized tomography (CT) scans, and photographs of brain after autopsy and fixation, in two cases with large arachnoid cysts. Case #1 is a male, 72 years old at death. This CT scan (**a**) was acquired at age 68. (**b**) The brain at autopsy following fixation from Case #1 shows the large left-sided arachnoid cyst cavity. Both the anterior temporal lobe and the orbitofrontal frontal lobe are affected by the lesion. Note that on the surface of the frontal cortex within the cyst cavity there is discoloration (white arrow). By contrast, the right side lacks evidence of indentation or contusion in the orbitofrontal cortex. Case #2 is a male, 64 years old at death. The CT scan (**c**) was acquired at age 56. As seen in the brain at autopsy following fixation (**d**), the arachnoid cyst is larger in the rostral-caudal axis in Case #2 in comparison to that of Case #1. However, the lesion is shallower—the affected region is smaller in the dorsal–ventral axis, and there was less impact on the orbitofrontal cortex (black arrow) in Case #2 compared to Case #1

*Case 1* A Caucasian male who was part of the University of Kentucky Alzheimer’s Disease Center longitudinal research cohort [10] came to autopsy at age 72 following a complex neurological history. His mother died with unspecified dementia, and his maternal grandmother had ALS. He worked in the field of clothing manufacture and retired at age 55. Progressive dementia was first noted at age 66, characterized by aggressive behavior that was clinically compatible with a frontotemporal syndrome. The subject’s history was also notable for epilepsy (complex-partial seizures), type II diabetes, depression, intermittent alcohol use (up to a 1L whiskey/week), and there was a clinical note of “post-Polio syndrome”. Reports of possible football-related concussions in high school were noted, as well as falls in late life, but more detailed information on closed head injury and brain trauma were lacking in the medical and research records. Final Mini-Mental State Examination score at age 70 was 17, final global clinical dementia ratings (CDR) scale score was 2, and final CDR “sum of boxes” = 13, indicating dementia of moderate severity. The patient died of acute cardiopulmonary arrest, unrelated to his dementia syndrome. Four years before death, a head computed tomography (CT) scan was performed as part of his seizure workup which revealed a large arachnoid cyst within the left temporal fossa and Sylvian region (Fig. 1a). Neurosurgical intervention was not pursued, in favor of observation. In serial CT scans performed over the subsequent years, the cyst remained stable in size, with progressive global brain atrophy notably increasing over time.

On post-mortem examination, the left side of his brain showed a large evacuated area measuring 75 × 50 × 30 mm (Fig. 1b). The lesion was adjacent to the left Sylvian fissure and resulted in extensive encroachment on the anterior temporal lobe, completely exposing the left middle cerebral artery from the ventral aspect (Fig. 1a, b). There was marked deformation and atrophy of the medial temporal lobe structures, and evidence for grossly apparent orbitofrontal subpial hemorrhage or contusion.

**Fig. 2:**
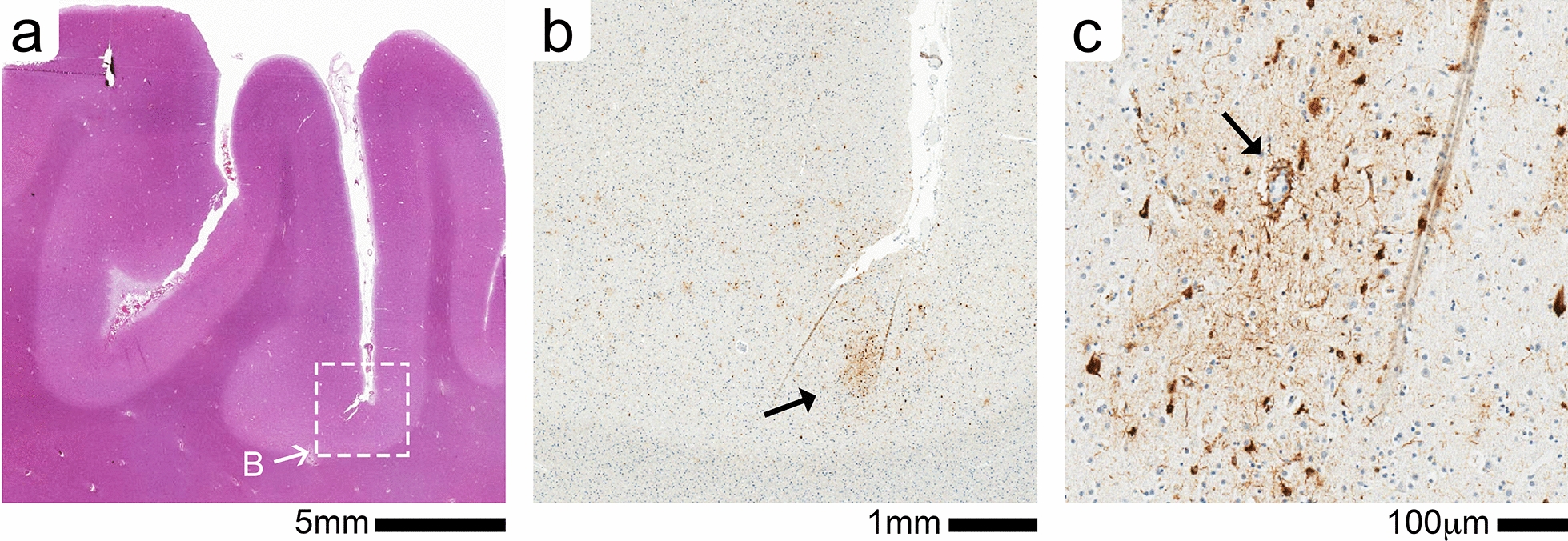
CTE-like neuronal pathology in frontal cortex (middle frontal gyrus) of Case #1. (**a**) Low magnification of H&E-stained section shows the location (white box) of the CTE-like perivascular cluster of phosph-tau (p-tau) illustrated in adjacent PHF1 + stained section (**b**). A single cluster of PHF-1 + staining seen in the depth of the cortical sulci (B, black arrow). (**c**) At a higher magnification the large vessel (black arrow) is surrounded by p-tau + neuronal and astrocyte staining

**Fig. 3:**
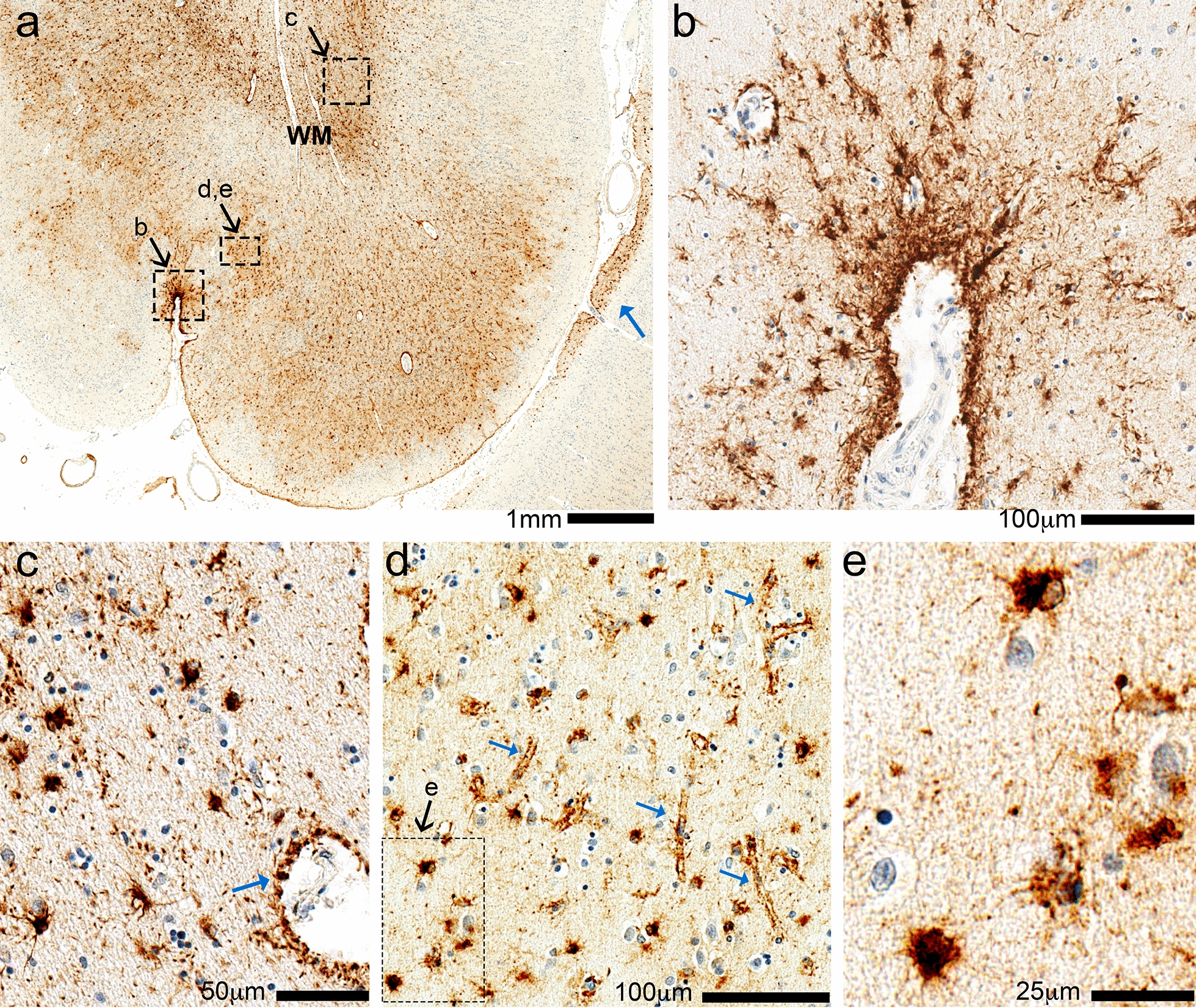
Phospho-tau (p-tau) immunohistochemistry in the left fronto-parietal region of Case 1. In the low-magnification photomicrograph (**a**), the pia mater is near the bottom and white matter (WM) near the top. This is a section of the left fronto-parietal cortex, which is caudal to the region most directly impacted by the arachnoid cyst, and shows widespread p-tau (PHF-1) immunoreactivity in the subpial, gray matter, and white matter regions. In each of these compartments there is prominent staining of p-tau surrounding small blood vessels, and also in cells with morphologic features of astrocytes. Subpial staining is demonstrated in a small sulcus (**b**) and additional subpial staining is shown at low power in the adjacent gyrus (blue arrow in **a**). (**c**) White matter shows p-tau cells with astrocyte morphology, as well as staining around blood vessels resembling astrocyte foot processes (blue arrow). (**d**) The astrocytic p-tau in gray matter highlight pericapillary staining (blue arrows); the inset shows compact p-tau + cells with astrocyte features (**e**)

Microscopic examination confirmed that the cyst lining comprised a delicate fibrous membrane with areas of an intact single layer of cells (arachnoid cells) and scattered foci of meningothelial cells. In the orbitofrontal cortex where discoloration (subpial hemorrhage) was noted on gross exam, the same area showed chronic degenerative changes including localized necrosis, neuropil rarefaction, and abundant hemosiderin-laden macrophages (not shown). A neurodegenerative assessment revealed an intermediate level of Alzheimer’s disease neuropathologic changes (ADNC): (Thal phase 5; Braak NFT stage IV; Frequent neuritic plaques). The 2012 NIA-AA consensus ABC score was A = 3, B = 2, C = 3. In addition, immunohistochemical stain for aberrantly hyperphosphorylated tau proteins (PHF-1 antibody [11]) displayed features of both CTE–NC (Fig. 2) and ARTAG–NC (Fig. 3). The CTE–NC-like changes included patchy distribution of neuronal PHF-1+ tau at sulcal depths of the cerebral cortex (Fig. 2). Several cerebrovascular pathologies were also identified, including small chronic ischemic infarcts.

While CTE–NC-like intraneuronal tauopathy was observed focally, the ARTAG–NC-like pathology was more extensive (Fig. 3). Numerous TSAs were seen within the white matter of the neocortex (frontal, temporal and parietal lobe) and the medial temporal lobe structures (amygdala, entorhinal cortex, and hippocampus). Many TSAs were present subjacent to the pia mater. The cortical regions adjacent to the arachnoid cysts showed prominent astrocytic tau accumulation arranged along small vessels at the depth of the sulci (Fig. 3d). The distribution and proximity of the CTE–NC and ARTAG–NC to the arachnoid cysts raised the question of whether this large space-occupying lesion induced the tau proteinopathy through chronic mechanical stress.

*Case 2* A Caucasian male was evaluated post-mortem by the University of Kentucky Pathology Department at age 64. He had previously been employed at a commercial nursery and used spray insecticides regularly on the job but retired before age 40. There was no known family history of dementia. Medical records indicated the presence of a “congenital” arachnoid cyst. His medical history was otherwise complex. He underwent a double lung transplant at age 57 years due to idiopathic pulmonary fibrosis, and was on tacrolimus. He had advanced liver disease with biopsy-diagnosed sarcoidosis, chronic CMV viremia (taking valacyclovir 900 mg twice daily) and rheumatoid arthritis treated with chronic steroids. At age 54, he “sustained a right-sided intracranial hemorrhage” and reportedly underwent craniotomy and surgical evacuation that left him with a partial facial hemiplegia. The arachnoid cyst was visualized at the University of Kentucky radiographically (shown in Fig. 1c) at age 56. Neurosurgical intervention was not pursued, in favor of observation. Serial CT scans performed at ages 58 and 62 years showed that the cyst remained stable in size. At age 60, he was seen by Neurology for parkinsonism, which was attributed to a medication (metoclopramide) side-effect. Concerns were raised about short-term memory loss at that evaluation, but cognitive impairment was never formally diagnosed. No other salient neurological symptoms were noted in the medical record review.

**Fig. 4:**
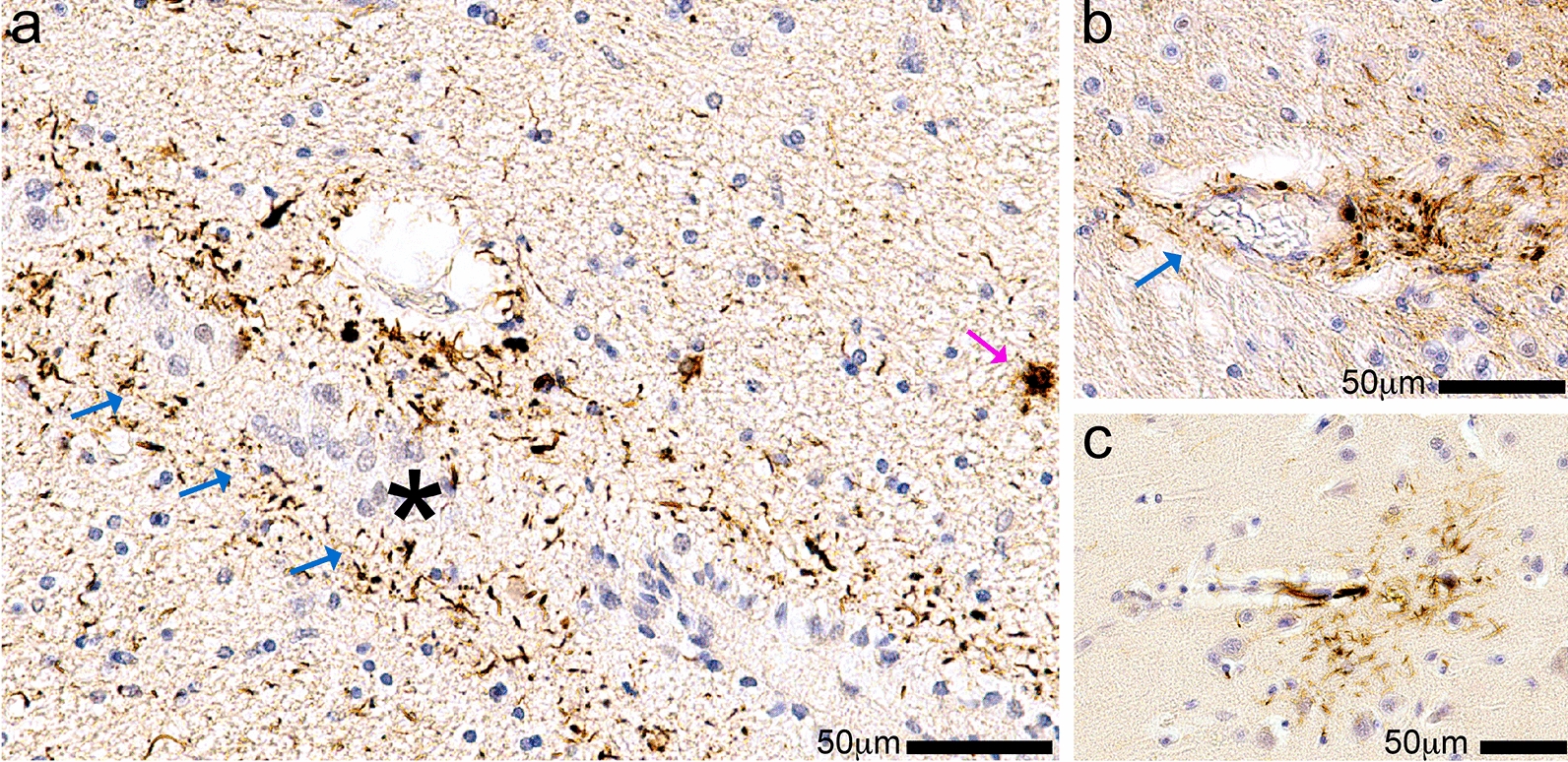
Sparse glial phospho-tau (p-tau) immunohistochemistry in Case 2. In this case, p-tau (PHF-1) immunoreactive structures were much more sparse, mostly non-neuronal, and were apparent in the medial temporal lobe. (**a**) In the hippocampus, p-tau immunostaining was found surrounding the rostral fringe of the inferior horn of the lateral ventricle (blue arrows; the ependymal lining denoted with an asterisk). Note that a few TSAs are stained (e.g., magenta arrow). (**b**) Shows p-tau immunoreactivity surrounding Virchow–Robin spaces of arterioles (blue arrow). Scattered p-tau + cells with delicate glial processes were also observed, such as that shown in (**c**) from the left-sided temporal cortex

On post-mortem examination, the brain showed an evacuated area on the left side measuring 80 × 35 × 30 mm (Fig. 1c, d). Compared to the arachnoid cyst seen in Case #1, this lesion did not appear as impactful on the orbitofrontal cortex, but still caused significant distortion of the temporal lobe. Microscopic examination confirmed the arachnoid nature of the cyst and revealed severe, widespread small vessel disease, including arteriolosclerosis, widening of the Virchow–Robin spaces, and perivascular pigment-laden macrophages. Sections sampled from near the arachnoid cyst were negative for AD–NC (neurofibrillary tangles and neuritic plaques); however, scant p-tau immunoreactive structures were focally present within the medial temporal lobe (Fig. 4). The p-tau immunoreactivity was seen adjacent to the ependymal lining of the lateral ventricle (inferior horn) (Fig. 4a) and occasionally surrounding Virchow–Robin spaces of the parenchymal arterioles (Fig. 4b).

## Literature review: astrocytes, brain injury, and tau protein

Clinical and biologic features of Cases #1 and #2 relate to three research domains: astrocytes; the sequelae of chronic mechanical stress and brain trauma; and tau pathobiology. Astrocytes are the most numerous cell type in the CNS, serving fundamental roles in support of brain function and regulating brain energy metabolism [12, 13]. Astrocytes also sustain synaptic activity and homeostasis [14], and contribute to CNS inflammatory signaling [15, 16]. The astrocyte glia limitans is a critical anatomic domain at the blood–brain-barrier and at the interface with the meninges [17]. Astrocytes also help remove cellular waste via glymphatic flow [18]. Given the many necessary functions of astrocytes in CNS physiology, astrocytic failure may contribute to synaptic dysfunction, neuronal loss, and neurodegeneration through a number of biologic mechanisms [19–21].

Astrogliopathy is a term used to describe a spectrum of astrocyte changes associated with injury or disease. Astrogliopathy can be divided into reactive astrogliosis or astrocytopathy [22], with the caveat that neither of these processes are thoroughly understood. The term reactive astrogliosis refers to astrocytic responses to changes in the environment. Following brain injury, reactive astrogliosis walls off the area of injury creating a functional border (“glial scar”) limiting molecular infiltration and recruitment of immune cells into the parenchyma [17]. With the proliferation of astrocytes and a loss of the astrocyte domain structure, a glial scar is an extreme example of a reactive astrocyte change, whereas many different alterations in brain physiology or disease may cause a milder reactive astrogliosis [22]. Reactive astrogliosis is typically both neuroprotective and reversible [22].

Astrocytopathy, in contrast, is a term which describes degenerative remodeling of astrocytes, and is linked to many different brain diseases. For example, Alexander’s disease is a primary astrocytopathy caused by the overexpression of the intermediate filament glial fibrillary acidic protein (GFAP) [22]. Although primarily linked to an astrocytic protein overexpression, Alexander’s disease is associated with misfolding of at least one other protein—TDP-43 [23]. ARTAG and severe (advanced-stage) CTE are astrocytopathies characterized by astrocytic tau inclusions.

The normal functions of tau protein are debated [24–26] and the roles for tau proteins in glial cells are mostly unknown. In the adult human brain, six tau isoforms are generated by alternative splicing of the microtubule associated protein tau (*MAPT*) gene*.* Depending on whether the proteins contain three or four carboxy-terminal tubulin-binding domains (“repeats”), the proteins can be classified as 3R or 4R tau, respectively [27, 28]. Immunohistochemical studies showed that astrocytes in ARTAG–NC and CTE–NC are negative for 3R tau and positive for 4R tau [29, 30].

To date, ARTAG–NC has not been reported in animal models in response to injury [31]. Yet it must be emphasized that rodents do not develop tauopathy in aging as humans do [32], and studies of genetically modified rodent models have primarily focused on neuronal tau [31]. The lack of astroglial tauopathy was mostly described in engineered mice with human tau transgenes driven by neuron-specific promotors that develop solely neuronal rather than glial tau inclusions. The animal models' inability to recapitulate human tau changes in response to brain injury could also be due to species difference in the native tau protein sequence, and/or kinases that contribute to the development of a tau astrogliopathy, as well as species differences in brain size and structure [33–36]. Thus, despite the similarities between the astroglial tau inclusions seen in CTE and ARTAG, there are few experimental model systems to study the pathology. The human phenomenology of tau astrogliopathies (as in other diseases) will likely prove informative. A goal of the present review is to discuss the relevant observations in human brains, that may aid in the development of mechanistic hypotheses for the development of tau astrogliopathy.

## Inter-relationships between CTE and ARTAG: evolving concepts

Associated with repetitive head injuries, CTE was first studied in boxers as dementia pugilistica or “punch-drunk syndrome”, with subsequent research on American football players and other elite athletes engaged in contact sports where closed head injury is common [37, 38]. According to the current criteria, the CTE–NC pathognomonic lesion is p-tau+ neurofibrillary tangles surrounding small blood vessels, in a patchy distributions at sulcal depths of the cerebral cortex [2, 39]. CTE–NC requires the presence of p-tau immunoreactive neurons with or without p-tau immunoreactive astrocytes; however, these lesions commonly coexist [2, 39, 40]. The distribution of tau pathology in CTE–NC and ARTAG–NC suggests some shared etiology [41–43]. Further, the presence of tau pathology in astrocytes in cases with CTE–NC suggests that ARTAG–NC could be a response of astrocytes to chronic mechanical stress or repetitive acute traumatic brain injury [1, 2, 43].

Whether a single moderate-to-severe TBI leads to CTE–NC is debated [1, 44–47]. Arena et al. [1] evaluated a series of cases with CTE–NC for astroglial tau and compared those cases to other tauopathies including ARTAG, AD, corticobasal degeneration (CBD), primary age-related tauopathy (PART), Pick’s disease (PiD), and progressive supranuclear palsy (PSP). Astrocyte tau in CTE–NC shared molecular properties with ARTAG cases. That is, the p-tau+ astrocytes expressed only 4R tau, similar to ARTAG-p-tau+ astrocytes, while neurons in CTE express both 3R and 4R tau, similar to AD [43]. Cherry et al. [48] examined 99 cases of CTE–NC for the presence of 3R and 4R tau. Neurons expressed both 3R and 4R tau pathology, while astrocytes only expressed 4R tau pathology. However, astrocyte tau changes were only found in the more advanced (Stage III/IV) CTE–NC cases. Intriguingly, p-tau+ astrocytes increased with age. A tipping point was seen in the sixth decade of life, with the percentage of p-tau+ astrocytes increasing linearly into the seventh and eight decade of life, at which point astrocytes accounted for at least 50% of the 4R tau+ cells in the perivascular lesions [48]. Many questions remain about the importance of intrinsic (including genetic) factors, injury physics, and the how idiosyncratic single-trauma (TBI) and multiple/repetitive trauma (CTE) conditions relate to these findings.

As awareness grows of CTE and its public health impact, case reports and cohort studies have accumulated outside the realm of contact sports [49–53]. These have included subjects with trauma from reported falls, vehicular accidents, military service, and other injuries that are independent of sports-related injuries. Interestingly, much of the reported pathology in such cases appears to be ARTAG–NC—perivascular p-tau+ astrocytes. However, there are also examples of p-tau+ neurofibrillary tangles at sulcal depths of the cerebral cortex in non-sports related CTE [2, 53].

In the two current cases with large arachnoid cysts, the individual (Case #1) with ARTAG–NC and CTE–NC like pathology had prior history of playing contact sports with documented concussions. In contrast, the person without ARTAG–NC or CTE–NC pathology (Case #2) had no known history of contact sports or TBI. These results support the connection between TBI and ARTAG but also suggest that TBI is not the sole driver of the ARTAG–NC seen in these cases. We cannot rule out that the nature of the arachnoid cyst in Case #1 was deeper and therefore itself caused added chronic mechanical stress related injury to the brain, as indicated by the bruising along the cyst cavity (Fig. 1). This hypothesis is supported by the findings of minimal/incipient ARTAG–NC in Case #2, that also demonstrated chronic mechanical stress related injury to the brain, without a history of acute TBI or closed head injury (Fig. 1).

The community-based autopsy study by Forrest et al. [54] provided insights into the prevalence of CTE and ARTAG in a high-quality European cohort. This study included 310 individuals aged 76-91 years old and evaluated multiple brain regions for the presence of CTE–NC and ARTAG–NC. One-third of the cases in this cohort were found to have ARTAG–NC. The findings of a very substantial proportion of elderly brains harboring ARTAG–NC are consistent with results from other community-based and clinic-based autopsy cohorts [30, 55–57]. It was also described that neocortical, as opposed to limbic or brainstem ARTAG–NC, was most strongly associated with dementia status [57].

Forrest et al. went on to explore the connection between a reported history of TBI and ARTAG–NC [54]. Of the 310 people included in the study, 67 people completed a self-reported TBI questionnaire. Only six subjects reported a loss of consciousness for at least 10 minutes (this number may reflect an underreporting or recall bias). If a single brain trauma caused ARTAG, ARTAG–NC would be expected in those six cases. However, only two of six cases with documented TBI were found to have ARTAG–NC—the same 1/3rd proportion as in the cohort overall. These results support the null hypothesis and underscore that more data is required about the relationship between clinical TBI history and ARTAG neuropathology.

In a novel approach to the study of brain trauma, Shively et al. [3] examined the brains of five individuals who underwent surgical leucotomy at least 40 years prior to death. The leucotomized subjects were schizophrenics and the study included matched subjects with schizophrenia that did not have leucotomy [3]. Thus, they controlled for severity, time, and location of the brain injury, while avoiding some of the recall bias inherent to sports and TBI studies.

In the leucotomy cases, as expected, pathological evaluation showed severe, localized white matter damage. CTE–NC was also present in some cases, including p-tau in neurons in cortical sulcal depths and around small blood vessels [3]. However, the dominant pathology was ARTAG–NC-like changes (astrocyte p-tau) and not neuronal CTE–NC pathology. The matched control cases lacked ARTAG–NC or CTE–NC changes [3]. Around the surgical lesion in the white matter, a dense astroglial scar was present along with subpial astrocytosis at the proximal depth of the overlying cortical tissue's sulci. Despite the severity of the subcortical white matter injury in the leucotomy cases, the gray matter was relatively intact; and cortical astrogliosis was absent. We note that Okamura et al. [58] also reported neuronal and glia p-tau pathology in 2 Japanese cases following leucotomy and provided a summary of previous neuropathological evaluations of leucotomy cases [58].

Shively et al. [3] tested whether tauopathy in astrocytes was a predictable component of glial scars or is a more idiosyncratic degeneration of a subset of astrocytes. Using serial sections, they stained for GFAP+ astrogliosis and p-tau. Despite the dense GFAP+ staining in the white matter adjacent to the cystic space-occupying lesion border, there was no p-tau staining in adjacent serial sections [3]. The lack of neuronal or astrocyte p-tau is informative as it shows that ARTAG–NC is not simply an unselective response to injury. Moreover, p-tau+ astrocytes were seen at the pia surface in depths of the sulci. In contrast to the intense astrogliosis seen at the border of the cystic space-occupying lesion, where ARTAG–NC was not seen, the subpial ARTAG–NC positive regions demonstrated only limited GFAP+ astrogliosis [3]. This suggests that astrogliosis alone was not the primary cause of ARTAG–NC. There must be other contributing factors, which may be specific to particular anatomic microdomains.

Shively et al. [3] suggested two hypotheses for the neuropathologic changes seen in their study. The first hypothesis was that axonal injury was the cause of the lesions. In the second hypothesis, the sulcal depths were more vulnerable to mechanical stress caused by the leucotomy procedure. Similar arguments for mechanical stress at sulcal depths have been proposed for CTE. The authors, however, stated their data supported the first hypothesis that the p-tau accumulation resulted from axonal damage. They further suggested that the accumulation of perivascular tau and the tau in the sulcal depths might be associated with the clearance of tau from the brain. They proposed that these locations were consistent with tau clearance via a glymphatic flux [3]. That is, the p-tau accumulation in the perivascular space and sulcal depths amounted to a ring around the drain as tau left the brain from injured axons. In our current case studies, it is notable that the arachnoid cyst in Case #2 caused long-term deformity of the brain, with far milder ARTAG–NC, underscoring that additional factors (intrinsic and environmental) may play important roles.

## Distinct populations of p-tau + astrocytes

Astrocytic tau inclusions are not only seen in ARTAG. Indeed, astrocytic tau changes can be a cardinal neuropathologic (if not pathognomonic) lesion in primary tauopathies, including tufted astrocytes in PSP, astrocytic plaques in CBD, globular astroglial inclusions in GGT, and ramified astrocytes in PiD [41, 42, 59]. Astrocytic tau changes in these primary tauopathies differ in morphology and location from those seen in ARTAG–NC. Importantly, as described in our case reports, there are subregions of the brain that appear more vulnerable towards the development of ARTAG–NC. Therefore, to understand possible causes of ARTAG–NC and how ARTAG–NC might alter brain physiology, it is important to appreciate the heterogeneity of ARTAG–NC.

**Fig. 5:**
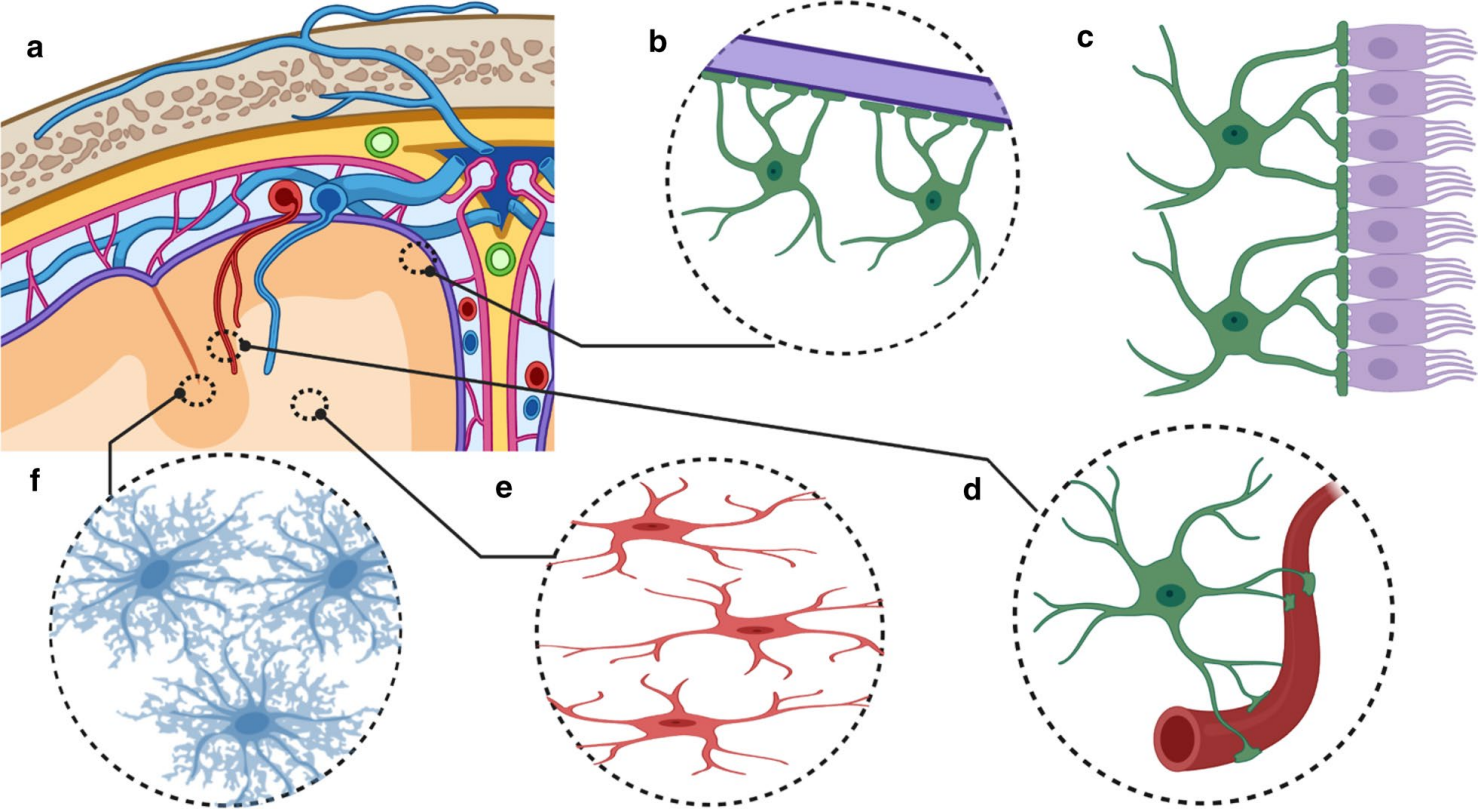
Illustration of morphology and location of aging-related tau astrogliopathy neuropathological changes (ARTAG–NC). (**a**) Phosphorylated tau protein (p-tau) immunopositive astrocytes can broadly be found in five parts of the brain. (*The illustration is not meant to be specific for any neuroanatomical region of the brain*)*.* TSAs are seen in the border-associated astrocytes of the subpial (**b**) and subependymal borders (**c**) (*location of C not shown on A*)*.* (**d**) Perivascular astrocytes are the third border associated TSA. (*It is not yet defined if these are exclusively arterial or are also venous associated astrocytes*)*.* (**e**) TSAs are also seen in the white matter. (**f**) In the gray matter, GFAs are the second disease-defining astrocyte morphology

ARTAG–NC may include three populations of border-associated astrocytes, as well as protoplasmic gray matter and fibrous white matter astrocytes (Fig. 5). The astrocytes' morphologic appearance defines the cells as TSA and GFA. In CTE and in response to brain injury, much of the ARTAG–NC is TSA. However, a different picture may be seen in neurodegenerative disease. Notably, individuals defined as healthy controls lacked gray matter ARTAG–NC, while 32% of AD/PART cases and 37% of Lewy body dementia cases had gray matter ARTAG–NC [60]. These results do not rule out the involvement of brain trauma as a contributor to ARTAG–NC. They do suggest that there may be heterogeneity among ARTAG subtypes, and it is important to consider differences in location and type of ARTAG–NC found.

As shown in Fig. 5, TSAs may be found in three border region-associated astrocytes, and some of the morphological features are shared across the different anatomical regions. This includes the TSA astrocytes at the subpial (Figs. 3b, 5b) and subependymal border (Fig. 5c). In these locations, the astrocyte processes of the TSAs reach the outer surface of the brain (Fig. 5b) or the central ependyma lining the ventricles (Fig. 5c). The subpial TSAs are likely marginal astrocytes, also known as interlaminar astrocytes. These are a relatively poorly studied population of specialized glia that form layers of endfeet at the pial surface, thus forming the glia limitans in the brain-meninges border. In humans, these marginal astrocytes send long, thin projections into the deeper cortical layers terminating in cortical layers II through IV [61]. Subpial TSAs are most often found in the cerebral cortical lobes, amygdala, hippocampus, basal forebrain, lateral midbrain, pons, medulla oblongata, and into the spinal cord [60]. Subependymal TSAs are seen in the third ventricle, the inferior horn of the lateral ventricle, aqueduct of the midbrain and medulla oblongata, and rarely in the anterior and posterior horn of the lateral ventricle [60].

The third border-associated ARTAG-vulnerable astrocytes are perivascular (Fig. 5d). The perivascular TSAs can be seen near the subpial surface, or in gray (Fig. 3e) or white matter (Fig. 3c) [60]. Subpial, subependymal, and perivascular astrocytes maintain blood-brain and brain-CSF-blood barriers, and regulate glymphatic flow [18]. If perivascular ARTAG–NC was abundant in the venous compartment, this could suggest a role in the movement of CSF and drainage out of the brain, whereas, if the perivascular ARTAG–NC was extensively in the arterial compartment, this could impact cerebral blood flow autoregulation. We identified ARTAG–NC surrounding Virchow–Robin spaces of arterioles (Fig. 4b) and surrounding capillaries in the cortex (Fig. 3d). Prior studies emphasized that ARTAG–NC can be found in the arterial compartment [41, 60]. More work is needed to evaluate ARTAG–NC associated with different cerebral vascular segments quantitatively, and if trauma versus neurodegenerative pathology causes a different vascular localization pattern.

**Fig. 6:**
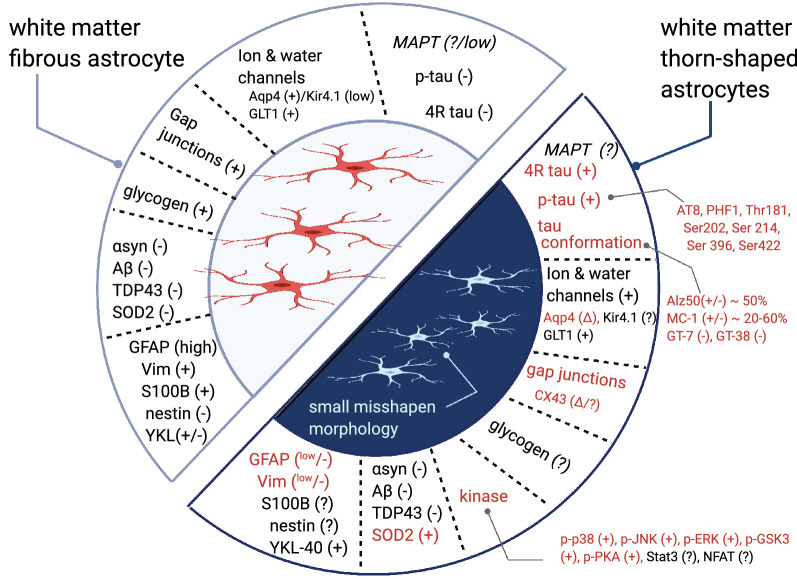
Comparison of white matter fibrous versus tau + thorn-shaped astrocytes. A summary of the molecular characteristics of healthy white matter fibrous astrocytes is shown in comparison to reported changes in white matter thorn-shaped astrocytes. The text highlighted in red are known changes in the thorn-shaped astrocytes in ARTAG

White matter is another compartment where TSAs are found in ARTAG (Figs. 5e, 6). The white matter of the medial temporal lobe has the highest likelihood of TSA, whereas the occipital lobe is the least likely [62]. Interestingly, the white matter of the corpus callosum, internal capsule, or cerebellar peduncles tend to be unaffected by ARTAG–NC [60]. The physiology of white matter fibrous astrocytes are poorly understood in healthy and disease states. By morphology, the fibrous astrocytes of the white matter have fewer branches than the gray matter astrocytes. The fibrous astrocytes align radially in the direction of the axonal tracks. These astrocytes also lack the tiling properties of gray matter astrocytes [61].

GFAs found in the gray matter are present most often in the amygdala, basal ganglia, cortex, and brainstem [63] (Fig. 5f). As the name suggests, GFAs have a fine granular appearance by p-tau-immunostaining, that appears to fill the cytoplasm of the protoplasmic astrocytes in the gray matter. GFAs have been found to be the most prevalent type of ARTAG–NC [54]. Further clues suggest that gray matter GFAs could be one of the earliest pathological changes associated with ARTAG–NC [54, 60, 62]. In our cases, GFAs were not widely observed in the cortical gray matter (Fig. 3d), suggesting that TSAs may be more specific to trauma, while GFAs could be associated with neurodegenerative pathology.

## Tau expression and protein modifications relevant to ARTAG

Little is known about tau expression (either transcription, or translation from mRNA) within human astrocytes. Via tubulin-binding domains, tau proteins may interact with microtubules, which are certainly present in astrocytes. Yet as far as we know, non-phosphorylated tau protein has not been visualized in quiescent astrocytes.

**Fig. 7:**
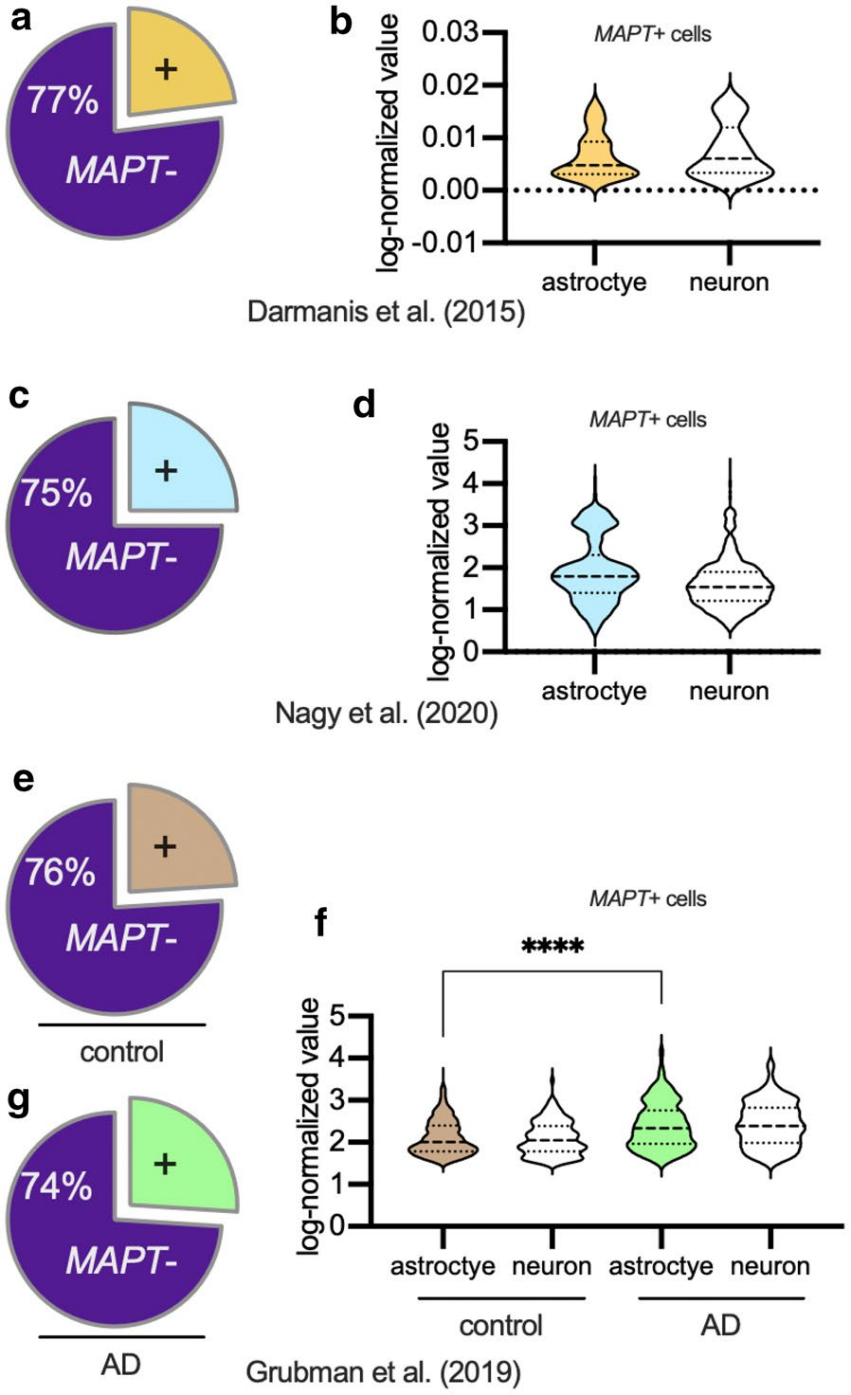
*MAPT* (Tau mRNA transcript) expression in astrocytes from human adult brain. Shown are results from three different single-cell and single-nucleus RNAseq studies (from outside laboratories) which analyzed mRNA transcripts in human brain samples. Pie charts (**a**, **c**, **e**, **g**) show that the proportion of astrocytes that express *MAPT* was remarkably similar (~ 1/4th of astrocytes evaluated express *MAPT* transcript) in all three studies. Among the astrocytes that expressed any *MAPT* (**b**, **d**, **f**), the amount of *MAPT* transcripts detected in astrocytes was approximately the same as in neurons in the same brains. In AD astrocytes that express *MAPT* (**f**), the level of *MAPT* transcripts detected was higher than in *MAPT* expressing control astrocytes

At least three studies reported single-cell or single-nuclei RNAseq data on human brain tissue suitable for studying *MAPT* transcripts in astrocytes. Their results were highly similar with each other in some important ways (Fig. 7). The first study conducted single-cell RNAseq on adult temporal cortex from individuals undergoing surgery for refractory seizures [64] (Fig. 7a, b). Using the authors’ cell type clusters, most astrocytes (77%) had low or nearly undetectable levels of *MAPT*. In comparison, 37% of neurons and 61% of oligodendrocytes had low or nearly undetectable levels of *MAPT*. On average, astrocytes expressed 67% less *MAPT* than neurons. Approximately a quarter of astrocytes express *MAPT* transcripts, and, notably, this population(s) of astrocytes express *MAPT* at similar levels to that of neurons. The second study completed single-nucleus RNAseq from the postmortem prefrontal cortex of 17 healthy control males as part of a study on major depressive disorder [65] (Fig. 7c, d). In these individuals, 75% of astrocytes expressed no *MAPT*. Yet, the astrocytes that did express *MAPT* again did so at comparable levels to neurons. The third study performed single-nucleus RNA sequencing from the postmortem entorhinal cortex of six AD patients and six sex- and age-matched controls [66] (Fig. 7e–g). Once again, approximately 3/4^th^ of the astrocytes did not express *MAPT*. Similarly, in the AD brain, 74% of the astrocytes did not express *MAPT*. In the astrocytes that did express *MAPT* transcripts, there was a significant (*p* < 0.001) increase in *MAPT* expression in the AD cases compared to control cases.

The single-cell and single-nucleus RNAseq studies were not informative about how the spatial localization of the astrocytes corresponded with *MAPT* expression. We also do not know if astrocytes upregulate *MAPT* when they become reactive. Neurodegenerative disease can increase astrocytic *MAPT* expression, suggesting that reactive astrogliosis could lead to astrocytes making more tau. However, the proportion of astrocytes that expressed *MAPT* didn’t increase with AD, indicating heterogeneity in astrocytes throughout healthy and diseased states. In summary, much remains unknown about *MAPT* expression in astrocytes including the basis for spatial heterogeneity of the *MAPT* expression.

Phosphorylation of tau is a widely studied tau posttranslational modification, and the pattern of phospho-specific epitopes expressed by ARTAG cells has been characterized [29, 43, 67]. Reactive astrocytes increase expression of some kinases [68, 69]. For instance, the mitogen-activated protein kinase (MAPK) are activated by stress, damage associated molecular patterns, and cytokines, and are known to be important pathways driving neuroinflammation and glial activation [70]. The MAPKs, among others, can also phosphorylate tau. TSAs were positive for the active (phosphorylated) form for all three MAPK pathways (ERK, JNK, p38), as well as GSK3B [29]. Ferrer et al. [67] completed phosphoproteomics on three control brain samples and three ARTAG brain samples. Interestingly, GFAP and aquaporin 4 were highly phosphorylated. They also found additional evidence for MAPK and cyclin-dependent kinases (CDKs) involvement from the phosphoproteomics [67]. These two studies imply that astrocyte activation may be driving high kinase activity in the astrocytes. This could be stimulating neuroinflammation, as well as phosphorylating tau. However, it has not yet been shown if the p-tau+ astrocytes express more kinase activity than the p-tau-astrocytes in brains with ARTAG–NC.

While they may be in some senses “reactive” to local tissue injury or insult, p-tau+ astrocytes have less GFAP staining and are smaller than p-tau-astrocytes from healthy control brains; they also appear to be misshapen [67]. Schwab et al. [71] explored a link between TBI and markers of cellular senescence in astrocytes. Specifically, they asked if a history of repetitive TBI increased γH2AX, a marker of double strand DNA breaks, in astrocytes. The increase in γH2AX was found in ependymal and subependymal cells and subpial astrocytes. This study did not specifically evaluate ARTAG, however [71]. While evaluation of white matter TSAs found the cells negative for ubiquitin, p62, beta-amyloid, and alpha-synuclein [29], perhaps suggesting that the astrocytopathy see was due specifically to tau misfolding. Of note, the p-tau+ astrocytes did express superoxide dismutase 2 (SOD2), and it was suggested that the SOD2 may be a cellular senescence marker [29]. It is unclear whether SOD2 expression contributed to or was a downstream effect of abnormal tau inclusions in TSAs.

Another theme that emerges from human phenomenology is the presence of glial tau inclusions at border regions between micro-domains and at connection points between cells. The molecular underpinnings of these observations are mostly unknown. Kovacs et al. evaluated glial aquaporin-4 and Connexin-43 in ARTAG cases [72]. Aquaporin-4 is a water-channel protein, expressed at astrocyte “endfeet”, which plays an important role in glymphatic function in the CNS [18]. Connexin-43 is a major gap junction protein expressed by astrocytes [73]. In areas with high astrocyte p-tau staining, connexin-43 and aquaporin-4 staining was increased or remain highly expressed [72]. The study did not specifically test whether the cellular localizations of connexin-43, and aquaporin-4 was altered. Overexpression and mis-localization of the proteins away from astrocytic endfeet might alter the physiological function of these proteins [18]. Further evaluation of these proteins and other endfeet proteins in ARTAG–NC is warranted.

## Pathologic mechanisms

Some of the potential disease-driving paradigms linked to ARTAG–NC derive from prior studies focusing on neuronal tau pathology. A growing body of evidence demonstrates that tau paired helical filaments (PHF) can promote further aggregation of tau in an amplifying, feed-forward mechanism similar to, or at least analogous to, prion diseases. In the nucleation-elongation mechanism, PHF tau “seeds” placed into the brain can initiate and accelerate more tau aggregation in an auto-propagating manner [27, 74]. This mechanism was supported experimentally using in vitro assays initially, but numerous studies have now demonstrated that preformed aggregated tau or human diseased brain extracts can accelerate tau aggregation in various animal models [75–77]. Here we focus on the studies most relevant to ARTAG–NC.

Tau seeds have been isolated from human brains with multiple tauopathies, including glial tauopathies. When injected into a mouse brain, tau seeds lead to trans-synaptic pathologic spreading that maintains the disease’s tau strain identity. For instance, AD tau extracts will seed neuronal tau, whereas PSP or CBD (human diseases with abundant glial tauopathy) brain extracts will seed glial tau [78]. In addition to the cell type specificity, the tau isoform from the diseased donor, be it 3R or 4R tau, were recapitulated in the recipient mouse: brain extracts from AD cases seeded 3R and 4R tau, while PSP and CBD brain extracts seeded 4R tau, and PiD seeded 3R tau [78, 79]. These studies showed that there are disease-specific, cell type-specific, and, to some extent, isoform-specific mechanisms underlying tau seeds.

Narasimhan et al. [80] asked if the formation of glia tau required neuronal tau. Using mice with a neuronal-specific deletion of *MAPT*, they tested if CBD-tau or PSP-tau could induce glial tauopathy. They found that CBD-tau injected mice had tufted astrocytes, and the PSP-tau injected mice had coiled body inclusions in oligodendrocytes [80]. This underscores that glia can form tau inclusions in the absence of neuronal-sourced tau. This study did not generate an astrocyte-specific *MAPT* knockout mouse to show that the aggregated tau was from endogenous astrocyte tau as opposed to some other cellular source.

Ferrer et al. [67] found evidence that ARTAG–NC derived tau seeds were unlike seeds sourced from other diseases, in that they did not maintain the disease phenotype. In this study, ARTAG–NC tau brain extracts injected in the mice resulted in p-tau in neurons, astrocytes, and oligodendrocytes. The tau did not maintain the 4R specificity, as the ARTAG–NC tau brain extracts seeded both 3R and 4R tau [67]. As ADNC is a common comorbidity with ARTAG–NC, there remains the possibility that the ARTAG–NC tau samples also contained some ADNC tau, and that the latter was biologically active in tau seeding experiments.

**Fig. 8:**
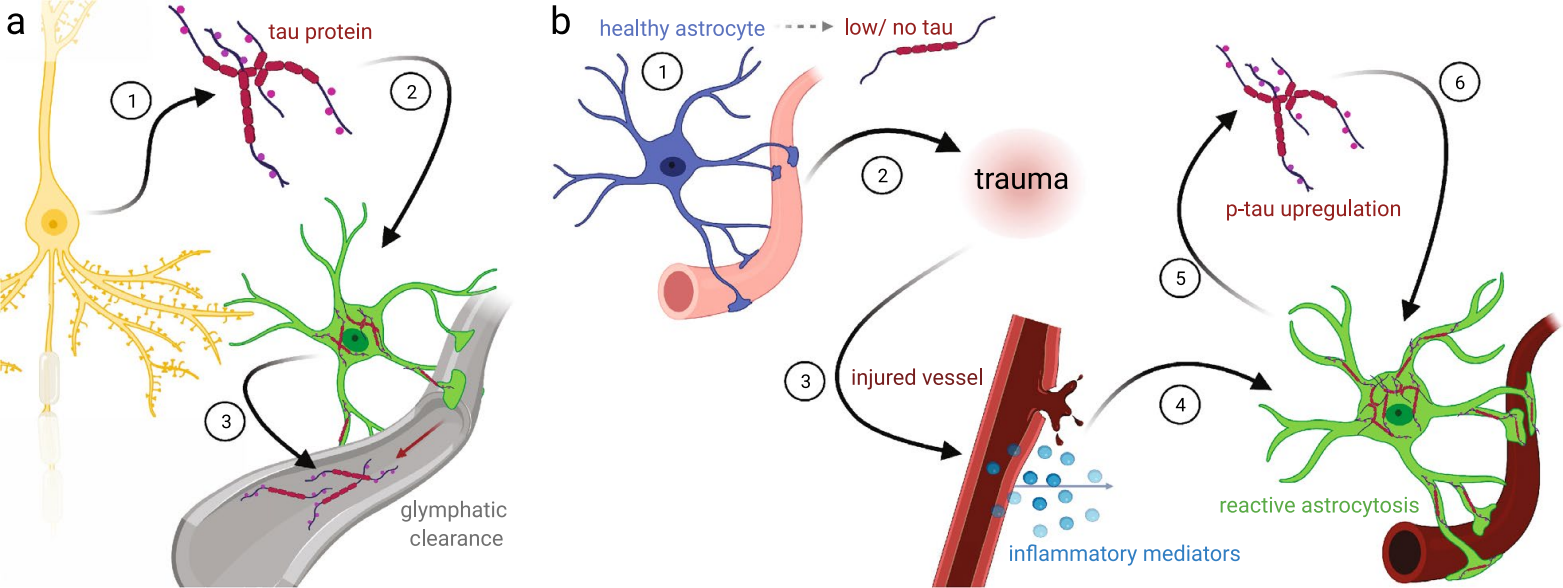
Two hypothesis of ARTAG development. (**a**) In the neuronal centric model, a neuron expresses the majority of the tau protein (1). The neuron releases this tau protein (2) which (after trauma or axonal breakage) may be taken up by an astrocyte (3) during the glymphatic removal of the tau protein. This leads to the accumulation of exogenous tau, and the seeding of aggregated astrocyte tau. (**b**) In the astrocyte centric model, most healthy astrocytes express no or very low levels of *MAPT*. Changes in the brain environment—from an injury, aging, disease, or a combination of factors—leads to a reactive astrocyte response (1), which may be centered around small blood vessels (2, 3). The reactive astrocytes then upregulate the expression of *MAPT* and increase kinase activity (4), which leads to the hyperphosphorylation and aggregation of tau (5), causing p-tau + aggregates in astrocyte cell bodies and perivascular foot processes (6)

The mechanisms leading to ARTAG are poorly understood. However, our review of the literature suggests two hypothetical models that are not mutually exclusive. In the first model, neuronal injury causes the release of tau protein seeds, which accumulate in astrocytes instead of (or perhaps while) being cleared from the brain (Fig. 8a) [3, 40]. This model assumes that the ARTAG–NC astrocytic p-tau is primarily of neuronal origin. As discussed above, ARTAG–NC is commonly seen in CNS border zones that are important for glymphatic removal of waste [3]. Hence astrocytes may take up tau released from cells, and the tau aggregates present in astrocytes may represent ingested remnants. A weakness of this hypothesis is that it does not explain why ARTAG tau pathology is exclusively composed of 4R tau.

A second hypothetical model proposes that brain injury, in combination with age, lead to a transformation of astrocytes in ways that promote ARTAG–NC. In this model (Fig. 8b), when astrocytes become reactive, they increase their expression of *MAPT* and have high kinase activity. It is also possible that non-translated *MAPT* mRNA could be translated in times of stress. The increase in tau and high kinase activity cause hyperphosphorylation and aggregation of tau within tau+ astrocytes. This model assumes that astrocytes—or at least a subset of astrocytes—make their own tau. As discussed above, ~ 25% of the astrocytes in the human brain express *MAPT* transcripts according to RNAseq experiments. In brains with neurodegenerative disease, astrocytes that express *MAPT* do so at higher levels than control brain astrocytes [66]. Moreover, there are many brains with ARTAG–NC that lack substantial neuronal tau pathology. Even in Case #2 in the present paper, we note that the minimal tau pathology observed was in glial appearing cells, within subependymal and perivascular microdomains. It is intriguing that this person, despite a very long-term lesion in the brain, albeit causing only mild mechanical stress, had minimal ARTAG–NC but also had undergone long-term treatment with strong anti-inflammatory drugs, given the possible role(s) played by inflammatory mediators in tauopathy. However, the complex medical histories of both these individuals underscore the challenges of direct cause/effect inferences that can be derived from human studies.

The limited number of astrocytes expressing *MAPT* could help explain the selective vulnerability of specific populations of astrocytes to ARTAG–NC and the patchy nature of ARTAG–NC. With chronological age, astrocyte morphology and molecular signatures are altered compared to young healthy brains, and astrocytes in aged brain respond differently to a TBI than astrocytes in young brains [20]. Further studies are required to explore the astrocyte subpopulations that express *MAPT* and their responses to brain injury.

We conclude that long-term space occupying lesions that impose chronic mechanical stress on the brain (in the examples described here, arachnoid cysts) may contribute to the development of ARTAG–NC in a stress dose-dependent manner. That is, while both cases demonstrate ARTAG–NC changes, Case #2 with less destructive mechanical stress was associated with mild and localized ARTAG–NC changes, whereas Case #1 with a more severe mechanical stress injury was associated with widespread and severe ARTAG–NC changes. In this manner, the case reports presented and the literature review suggest that CNS trauma (chronic or acute mechanical stress) can drive ARTAG–NC-like pathology. The lesions’ morphologies and locations suggest that the ARTAG–NC-like brain changes following injury from mechanical stressors differs from that seen with neurodegenerative diseases. More work is required to test models of ARTAG–NC development and the possible interaction of seeding and tau aggregation models. Future work will also be needed to understand the normal physiological function of tau phosphorylation in astrocytes (if any), and how ARTAG–NC changes astrocyte physiology. As we learn more about ARTAG, we will gain a greater understanding of how astrocytes contribute to the long term sequelae of CNS trauma and age-related cognitive decline.

## Data Availability

All data generated or analyzed during this study are included in this published article.

## Abbreviations

ADNC: Alzheimer’s disease (AD) and neuropathologic changes
ARTAG–NC: Aging-related tau astrogliopathy (ARTAG) and neuropathological changes
AP: Astrocytic plaques
CBD: Corticobasal degeneration
CDKs: Cyclin-dependent kinases
CTE–NC: Chronic traumatic encephalopathy (CTE) and neuropathological changes
CT: Computerized tomography
GFAP: Glial fibrillary acidic protein
GAI: Globular astroglial inclusions
GGT: Globular glial tauopathies
GFA: Granular-fuzzy astrocytes
LBD: Lewy body dementia
*MAPT*: Microtubule associated protein tau
MAPK: Mitogen-activated protein kinase
PHF: Paired helical filaments
PART: Primary age-related tauopathy
PiD: Pick’s disease
PSP: Progressive supranuclear palsy
p-tau: Phosphorylated tau protein
RA: Ramified astrocytes
SOD2: Superoxide dismutase 2
TBI: Traumatic brain injury
TSA: Thorn-shaped astrocytes
